# The Impact of the COVID-19 Pandemic on Research and Volunteering Activities among Medical Students: A Cross-Sectional Study among Romanian and International Students from One Medical Faculty from Romania

**DOI:** 10.3390/ijerph19127477

**Published:** 2022-06-18

**Authors:** Andreea Iulia Pop, Simona Mirel, Mira Florea, Lucia Maria Lotrean

**Affiliations:** 1 Discipline of Hygiene, Department of Community Medicine, Iuliu Hatieganu University of Medicine and Pharmacy, 400012 Cluj-Napoca, Romania; llotrean@umfcluj.ro; 2 Discipline of Medical Devices, Department 4 Pharmacy, Iuliu Hatieganu University of Medicine and Pharmacy, 400012 Cluj-Napoca, Romania; 3 Discipline of Family Medicine, Department of Community Medicine, Iuliu Hatieganu University of Medicine and Pharmacy, 400012 Cluj-Napoca, Romania; miraflorea@umfcluj.ro

**Keywords:** COVID-19, pandemic, research, volunteering, medical undergraduate students, Romania

## Abstract

Introduction: This study focuses on the influence of the COVID-19 pandemic on medical students in their last year of study at the Faculty of Medicine of the University of Medicine and Pharmacy in Cluj-Napoca, Romania. It analyzes the impact of social distance limits on the conduct of research activities required for the development of the graduation thesis, as well as assesses sixth-year students’ attitudes and behaviors toward volunteer activities during the COVID-19 pandemic. Methods: A cross-sectional survey was conducted in June–July 2021 among sixth-year students. It used an anonymous online questionnaire and was sent to all students from the Faculty of Medicine studying in the Romanian, English, or French sections (*n* = 606). The response rate was 38.28%, resulting in a final sample of 232 students from both Romanian and international sections. Results: The findings demonstrate that two-thirds of the students in the survey agreed that the COVID-19 pandemic had a substantial impact on their research. Some of the students had to choose another subject for their graduation thesis, while about a quarter of the students gave up collecting personal data, 25% of respondents stated that it was difficult to contact research participants, one-third of them linked the difficulty of doing activities at the hospital to the present outbreak, and almost 30% of the students had difficulty communicating with the coordinator. The disturbance of the research activity seems to be higher among international students in comparison with the Romanian students, while there are limited gender differences. One out of five medical students has been involved in volunteer activities during the pandemic. Most have opted to volunteer at hospitals (especially international students) and contribute to health education activities (especially women). Conclusions: This is the first study from Romania and one of the few from Europe that presents data regarding the influence of the COVID-19 pandemic on research and volunteering activities among medical students.

## 1. Introduction

Since the beginning of the epidemic, the SARS-CoV-2 virus (Severe Acute Respiratory Syndrome Coronavirus-2) has infected over 250 million individuals around the world, causing severe acute respiratory syndrome and killing over 5 million people. In Romania, around 1.7 million people have been infected with the novel coronavirus, resulting in over 50,000 deaths [1].

With the onset of the global COVID-19 pandemic, urgent political decisions were required to limit the spread of the new coronavirus infection, resulting in the implementation of some non-pharmaceutical interventions, such as reducing contact between individuals as much as possible, protecting vulnerable people, closing educational institutions, suspending events, closing restaurants, reducing employee working hours, allowing employees to work from home, and restricting travel [2,3]. The introduction of new global social distancing policies in the early months of 2020 has pushed most medical schools across the globe to complete practical courses and activities in the virtual environment via online platforms. The following factors influenced this decision: increased risk of viral transmission, fear of interaction with asymptomatic carriers, and a lack of protective equipment [4,5,6].

Various challenges in the educational process have been noted as a result of the transition to a virtual setting. On the one hand, many students were distracted during class due to their family environment. In their study, Arowoshola provided a different perspective more relevant to this situation, which followed student parents who needed to focus entirely on teaching or childcare while still attending to their medical education because of the lockdown of all educational institutions [7]. Online meetings have also been disrupted based on inaccurate internet access or user faults. Additionally, all students were required to have the necessary equipment (media, software/applications, internet access, and access to information platforms) to participate in study programs [8,9]. The shock of this abrupt transition forced the entire academic community to become active in the optimal operation of the online educational option. E-learning improves the educational program’s efficiency and flexibility by virtually eliminating travel time between face-to-face lessons [4]. Additionally, media podcasts and specific teaching applications, a variety of teaching strategies, and access to various educational databases, some of which are free and available via university libraries, can have a positive impact on students by stimulating the motivation to acquire new information, according to scientific studies [6]. 

The World Federation of Medical Education (WFME) has promoted the scientific method as one of the fundamental principles for undergraduate medical education, while the Association for Medical Education in Europe (AMEE) encourages students to participate in individual research projects [10]. 

The COVID-19 pandemic has had a significant impact on current educational research. While conducting a study, educational researchers meet numerous challenges. To protect study participants and research staff, many academic medical institutes have suspended research to eliminate non-essential contact. As the pandemic impact on the healthcare system grows, research staff have been reassigned to other tasks. COVID-19 infection among scientific members and participants has resulted in a reduction in the number of researchers in the field. In the case of the COVID-19 pandemic, the focus of research has relocated to online research via Google forms, voice calls, and video calls. Because the COVID-19 pandemic is such a pressing public health issue, the focus of research, in general, has shifted to COVID-19 -related research. There are funding constraints, and most of the available research funds have been shifted to COVID-19 research [11]. Except for COVID-19-focused studies, most clinical trials were suspended [12]. The COVID-19 pandemic’s restrictions have had a significant impact on researchers all across the world [13].

In terms of university medical education in Romania, the effects of e-learning may also be seen in the research activity that students must do in their final year as part of their graduation thesis. Students are required to take an examination at the end of the sixth year that consists of a theoretical test, a clinical test, and an assessment in which graduates present a thesis based on data collected through a scientific study performed under the supervision of a teacher. This thesis is divided into two parts: the first contains data from the literature on the subject of the graduation thesis, and the second presents the graduate’s research findings. Because of the restricted interaction with the medical environment, data collection, patient interviewing, access to medical documents, and laboratory analyses have become almost impossible to execute. 

The medical system’s crisis, which began with the outbreak and has resulted in overburdening medical institutions, has prompted the introduction of new initiatives to improve medical service provision internationally. In one study, Soled et al. mentioned that the medical profession is facing one of its greatest challenges and needs to act. Advanced medical students have skills and practical knowledge that they may use to help doctors, staff, and patients, but they are limited in their potential as students [14]. The British Medical Association highlighted that students should not be encouraged to “step up” and act in situations when they are not qualified [15]. Many medical colleges throughout the world launched student volunteer initiatives in response to the COVID-19 epidemic [16]. The concept of student involvement during a crisis is not unusual; during the Spanish flu pandemic in 1918 in Pennsylvania and the Polio outbreak in Copenhagen in 1952, students helped doctors [17].

Several studies have described the use of digital technology to improve access to medical treatments for chronically vulnerable individuals while preventing iatrogenic COVID-19 infection. Student volunteers from Columbia University in New York worked with outpatient clinics to enroll patients in institutional telehealth systems in order to minimize the number of in-person visits while maintaining patient access to their healthcare professionals [18,19,20]. The measures of social separation and the discontinuation of academic activities had a significant impact on the educational process for students in their last year of study. Participation in clinical activities in medical schools has varied across states. Some faculties have chosen to adjust the curriculum, facilitating video conferences, while others have reduced the number of hours spent in hospitals. In countries such as the United Kingdom and Italy, it has been decided to accelerate efforts to graduate students in the last year to supplement the workforce in the overworked health system [21,22,23]. Concerned about the medical breakdown that was overtaking the entire society at the start of 2020, during the outbreak of the new COVID-19 pandemic, medical students were strongly encouraged to participate in volunteer activities. Thus, medical students from several medical schools have participated in a variety of community health education initiatives, cooperating with medical staff to monitor the condition of COVID-19 infected patients. Some were assigned to work in hospitals, while others helped with the delivery of food and medicine to the elderly or vulnerable people [24,25,26]. In the United States, medical students at Washington University in St. Louis formed The Washington University Medical Student COVID-19 Response (WUMS-CR) to assist with the epidemic around the country [27,28]. Student leaders at Harvard Medical School (HMS) formed a COVID-19 Medical Student Response Team to build a student organization that would improve students’ ability to efficiently mobilize interested colleagues in the COVID-19 response, both clinically and in the community, as well as to identify evolving needs and rapidly engages students in the response [14]. Teachers and student leaders at Columbia University in New York formed the COVID-19 Student Service Corps (CSSC), which aims to provide interprofessional student service-learning projects to health systems dealing with the COVID-19 epidemic, including patients, workforce, and communities [20]. In Europe, in response to this pandemic, the Poznan University of Medical Sciences in Poznan, Poland (PUMS), created a COVID-19 student volunteering program in partnership with a student volunteering project (SVP) [16]. In Germany, non-final-year students were encouraged to volunteer at healthcare institutions by the German Minister of Health; the final-year students were treated differently in various hospitals [23]. Another research found that medical students at Aalborg University in Aalborg, Denmark, were key members of the pandemic emergency healthcare workforce during the COVID-19 pandemic [29]. During the pandemic, medical students at the Cluj-Napoca University of Medicine and Pharmacy were encouraged to participate in hospital activities, public health directorate activities, patient follow-up, health education activities, delivering medicines and food to isolated or quarantined people, and working in vaccination institutions.

This study focuses on the influence of the COVID-19 pandemic on Romanian and international students in their last year of study at the Faculty of Medicine of the University of Medicine and Pharmacy in Cluj-Napoca, Romania, and has three objectives. First, it evaluated the impact of the pandemic on the conduct of research activities required for the development of their graduation thesis. Second, it assessed sixth-year students’ attitudes and behaviors toward volunteer activities during the COVID-19 pandemic. Last, but not least, the influence of gender and the study section (Romanian vs. international section comprising the English and French sections) on the investigated behaviors was investigated.

## 2. Materials and Methods

### 2.1. Study Sample and Procedure for Data Collection

This study is part of a research project focused on the assessment of the involvement of medical students in research and volunteering activities, which received the Ethical Approval of Ethics Commission of Iuliu Hatieganu University of Medicine and Pharmacy (Approval no. 194/19.04.2018). 

The first wave of the study was a cross-sectional survey conducted in 2018, while the second wave of the study was carried out in June–July 2021.

This paper presents the results collected during the second wave of the study among sixth-year students from the Faculty of Medicine of the University of Medicine and Pharmacy Iuliu Hatieganu from Cluj-Napoca, Romania.

The university’s academic educational offer includes opportunities for learning in the medical faculty for both Romanian students in study programs with teaching in Romanian and international students, citizens of the European Union, the European Economic Area, the Swiss Confederation, or third countries in study programs with learning in English and French. 

All students in their last year of study, a total of 606 students, 332 from the Romanian section, 167 from the English section, and 107 from the French section, were invited to participate in this study by filling out an anonymous online questionnaire. The students received an invitation that explained that the participation is voluntary, and by filling in the questionnaire, they accepted the participation. People who did not want to participate did not fill in the questionnaire. 

The response rate was 38.28%, resulting in a final sample of 232 students. Thus 64%, which means 149 students, were in the Romanian section, and 36%, 83, were international students (20% signifying 46 students in the French department, and 16% representing 37 students in the English department). Of the respondents, 59% were girls, while 41% were boys. Along these lines, in the section with teaching in Romanian, 36.9% were boys, and 63.1% were girls, while in the department with learning in English and French, 49.3% were boys, and 50.6% were girls.

### 2.2. Instrument for Data Collection

The questionnaire investigated several issues regarding the involvement of medical students in research and volunteering activities. Moreover, in 2021 we investigated how students perceived the impact of the COVID-19 pandemic on the research effort necessary for the graduation thesis, as well as their involvement in voluntary activities during the pandemic. 

We assessed the extent to which the pandemic influenced the preparation of the students of the research thesis by asking them which of the following impacts were perceived by them concerning changing the subject of the thesis, abandoning the collection of personal data, and conducting a review of the literature data, the effect of the pandemic on contacting study participants and data collection, the difficulty to develop some activities in the laboratory, in hospital, or in the community. In addition, we asked if centralization and the interpretation of data were affected, as well as communication with the coordinator of the graduation thesis.

Moreover, we wanted to know if the students participated in any community services during the COVID-19 epidemic (March 2020–May 2021). We wanted to know what types of activities medical students have performed, so we offered the following as options: volunteering in public health directorates, in-hospital institutions, patient follow-up in collaboration with general practitioners, volunteering in the field of transporting medicines or food for citizens in isolation/quarantine, volunteering in vaccination institutions, and health education activities.

### 2.3. Data Analyses

The prevalence of the investigated issues was calculated for the whole sample as well as separately for the Romanian section and international section, comprising English and French sections. A score of the intensity of disturbance of research activity among students was calculated by summing the presence of different problems (0—no, 1—yes, the maximum number of investigated problems was 8).

Logistic regression was used to assess to what extent the study section or the gender influenced the investigated issue.

Linear regression analyses were used to assess if there were differences between the two sections or between women and men concerning the score of the intensity of disturbance of research activity among students.

The data were analyzed using SPSS 22 statistical package, and significant results are reported at 0.05.

## 3. Results

The findings demonstrate that two-thirds of the students in the survey agreed that the COVID-19 pandemic had a substantial impact on their research. When asked how they intervened in the research, 9% of respondents said they had to change the subject of their thesis, while 23% said they had to review the literature instead of data collection. A quarter of those polled said the current epidemic made it difficult to contact study participants and hence collect data. The respondents indicated that the health crisis made it difficult to develop research activities in the laboratory in 12% of cases, 35% believe it made it difficult to develop research activities in the hospital, 6% of students believe that the pandemic interfered with school activities or community activities, and students believe it made it difficult to centralize and interpret the results in 14% of cases. Furthermore, a third of students believe that communicating with the coordinator was difficult during this time (see Table 1).

As presented in Table 2, when asked if they were involved in volunteering activities during the outbreak around March 2020–May 2021, the majority of students said no, while 22% said they did. Thus, about 6.9% were involved in hospital activities, 2.6% volunteered in the public health directorate, and 3% assisted in patient follow-up in collaboration with the general practitioners. Volunteering students carried out health education activities in 5.2% of cases, assisted deliver medicines and food to isolated or quarantined people in 3.4% of cases, and worked in vaccination institutions in 2.2% of cases.

As presented in Table 3, the logistic regression findings show that the impact of the COVID-19 pandemic was more strongly perceived by international students concerning the need to perform a review of the literature instead of data, as well as with the possibility to perform research in the hospitals. On the other hand, female students tended to report more problems regarding data centralization and analyses. Moreover, the results of the linear regression analyses showed that the score regarding the intensity of disturbance was higher among international students (standardized beta = 0.170, CI = 0.130–0.928, *p* < 0.010, but no gender differences were found about this issue (standardized beta = 0.035, CI = −0.288–0.500).

Regarding volunteering activities, the logistic regression findings show that participation in volunteering hospital activities was higher among international students. They also indicate that female students were more involved in health education activities during the COVID-19 pandemic. No gender differences or differences based on the studying section were observed in the other investigated issues related to volunteering activities (see Table 3).

## 4. Discussion

This study provides data on the impact of the COVID-19 pandemic on Romanian and international students in their final year of study at the Faculty of Medicine of the University of Medicine and Pharmacy in Cluj-Napoca, Romania.

Our findings relate to the effect of the current health crisis on the proper conduct of research and volunteering activities performed by medical students. Several international studies have documented the impact of the COVID-19 pandemic on medical education, following the implementation of social distancing measures to avoid the virus’s spread [4,6,30]. Concerns surrounding academic performance have been raised as a result of adopting virtual pedagogical practices. The majority of studies concentrated on the impact of the absence of clinical rotations in hospital settings on the development of clinical skills [8,9]. New educational approaches, such as telemedicine, have emerged as a result of the necessity to identify new strategies to facilitate interaction between students and patients [31]. Moreover, most universities had to interrupt typical clinical scientific studies and restrict access to laboratory research environments due to infection control efforts [10].

Several publications have explored the impact of excluding future graduates from healthcare institutions and conducting online medical education [23]. To the best of our knowledge, no previous study from Romania has investigated the impact of the pandemic on the involvement of students in research activities.

Medical students’ education and participation in research activities differ between countries and universities [32]. Students of the Faculty of Medicine of the University of Medicine and Pharmacy in Cluj-Napoca are encouraged to participate in research activities from the beginning of their studies. A previous study focused on undergraduate medical students’ attitudes, and practices toward research highlighted positive attitudes, motivation, and interest in this field. They have a module on scientific research techniques in their second year of study, in which students are trained on how to evaluate medical research critically and about principles of medical science. The final exam at the end of the medical studies course includes a component for preparing a research thesis. Following that, the thesis is presented in front of a committee and earns a score that accounts for half of the summative assessment on the graduation test [32]. 

The graduation project production was affected by the COVID-19 pandemic. Two-thirds of students stated that legal constraints had an impact on them. Specific questions about conducting research during the pandemic were asked by Steven J. Elmer and John J. Durocher in their editorial in which they offer suggestions for moving student research forward during the COVID-19 pandemic: “ (1) Can the original research question still be addressed? (2) Are the hypotheses still testable? (3) Can the experiments be carried out safely? (4) Could modifications be made to the experiments to improve safety in regard to COVID-19, and (5) Will the research be completed if there are future COVID-19-related interruptions?” Based on the responses to these questions, it is crucial to see if the original study topic and hypotheses can still be treated by “adapting the methodology” to fit within COVID-19′s limitations [33]. 

Thus, in our study, some of our responders noted that they were even forced to abandon the theme they were initially chosen. Almost a quarter of participants stopped gathering personal information and instead performed literature review research. Similarly, 25% of students had difficulties in contacting research participants and gathering data. Furthermore, one-third of the students stated that it was difficult to perform research activities at hospitals, which may be attributed to the overloading of these institutions as a result of the huge number of patients contaminated with the novel coronavirus. As a result, access to the medical documents from the archive, as well as communication with patients with chronic disease became considerably more difficult [34,35]. 

The preliminary findings of a survey sent to undergraduate students from 17 different universities in the United States who researched or planned to research during the COVID-19 epidemic revealed that the undergraduate students were affected by the pandemic. Most researchers who were performing studies in Spring 2020 continued to do so. However, there were some interruptions due to the epidemic. Students were faced with a variety of situational obstacles, including the inability to meet with teams and mentors in person, inadequate connection to the internet, time pressure, and missed opportunities to present their findings. About half of those who planned to perform research during the summer semester was forced due to the pandemic to cancel their plans. Students who were still able to do research during the summer semester faced challenges such as a lack of face-to-face discussions with their research teams, uncertainty about their research projects’ subsequent phases, and finding motivation in the middle of the outbreak [36]. Linked to previous findings, about a third of students in our study declared that the present pandemic has made it more difficult to communicate with the thesis coordinator, probably due to the overloading of teaching and medical activities of the supervisor. According to 12% of the students, the performance of research activities in the laboratory has also been affected, presumably due to the orientation of all research towards the novel coronavirus or the temporary shutdown of research institutes [12]. According to a survey conducted by the American Physiological Society, 58% of students believe that laboratory closures caused by the pandemic will extend the time required to finish their studies [33]. The closure of public schools and distance restrictions, for some of the participants, interfered in the conducting of research activities in the school or the community. The disturbance of the research activity seems to be higher among international students in comparison with the Romanian students, probably because international traveling had several restrictions, and students from international sections spent most of the time between May 2020 and February 2021 in their own countries and participated in online teaching and training activities. On the other hand, there are limited gender differences concerning the effects of a pandemic on research activities of students, with both women and men being affected. 

According to evidence from another study focused on the impact of the COVID-19 pandemic on scientific research, the pandemic has affected academics in all fields of research. Due to university and research institution closures, researchers may not finish the fundamental experiments necessary for the publication of scientific articles and may feel insecure about releasing knowledge online at scientific conferences [13]. Several clinical trials were suspended, except those focusing on COVID-19. Alternatively, several active clinical studies, as well as those on the waiting list, have been modified to allow for the use of virtual monitoring systems to guarantee the safety of research participants [37].

In one survey, academic staff from 31 Irish and UK medical schools were questioned about the pandemic’s impact on undergraduate research instruction and project possibilities, as well as actions made to reduce the pandemic’s effects on these curricular aspects. According to over 75% of respondents, COVID-19 has a negative impact on the availability of clinical research, lab-based research, and summer research programs. The authors enhanced some novel strategies such as adaptation and adjustment in research project design, relocation to online platforms, research dissemination in the virtual environment, increased workload for research program delivery staff, flexibility around assessment of research-based assignments, and value of open communication [10].

The COVID-19 pandemic’s consequences on the worldwide medical system, as well as the move of all medical education to the virtual environment, have prompted some medical schools to consider innovative approaches to health education and treatment. Some institutions have advocated early graduation choices for students in their last year of study, while others have started and supported volunteer initiatives to help the overcrowded healthcare system [16,23,38]. From another perspective, when traditional educational programs were interrupted, the involvement of students in volunteering permitted them to develop skills [27].

According to our findings, about 22 percent of the students in the final year at the Faculty of Medicine of the University of Medicine and Pharmacy were involved in community services during the COVID19 pandemic. Approximately 7% said they helped at the hospital, while about 3% agreed to help public health directorates and general practitioners follow up on patients. Research on the role of medical students during the COVID-19 pandemic encourages student participation in outpatient consultations, in the non- COVID-19 medical team, and also in phone consultations, which are required to monitor the health of infected patients with the novel coronavirus at home [39]. About 5% were active in health education groups, just 2.2% worked in vaccination institutions, and 3.4% of students offered to bring medications and food for isolated or quarantined patients. Similarly, a report on medical education during COVID-19 details medical students’ engagement in community service, including food collection, population education, house monitoring of quarantined COVID-19- positive patients, and involvement in delivering food or medicine across Chicago [24]. 

Several international studies have also shown medical students’ participation in volunteer activities during the pandemic, focusing on various areas to respond to the needs of the medical system and the community. Considerable efforts of the Washington University Medical Student COVID-19 Response (WUMS-CR) aided the Washington University and St. Louis communities. During the first three months of operation, students at the Washington University School of Medicine recorded over 12,000 volunteer hours. WUMS-CR’s volunteer efforts amounted to around 24 staff members working full-time (40 h per week) over three months. Childcare for healthcare professionals, daily literature summaries of novel research delivered to medical staff, and a student-run triage were among the first volunteer programs introduced. Other requirements developed subsequently, such as the fabrication of face shields and masks [27]. At the Harvard Medical School, the COVID-19 Medical Student Response Team had over 500 medical student volunteers in a very short time and had shared the response team’s organizational framework with several medical schools around the country. The student volunteers joined one of four virtual committees: “Education for the Medical Community, Education for the Broader Community, Activism for Clinical Support, and Community Activism” [14]. 

Faculty and student leaders at Columbia University in New York collaborated to develop the COVID-19 Student Service Corps (CSSC) service-learning projects, which include over 1600 student volunteers working on almost twenty projects. Volunteering students collaborated with clinics to introduce patients to institutional telehealth platforms, developed materials for a variety of audiences, and supported organizations with food delivery and other essential resources. Students were also involved in the creation of a personal protective equipment task force to manage PPE acquisition, donation, and production [20]. 

Within 2 weeks of mobilization, undergraduate students from Aalborg University and Aalborg University Hospital in Denmark and all master’s students had volunteered, and almost two-thirds of students were working as temporary residents 47%, ventilator therapy assistants 28%, or nursing assistants 19%. More than 70% of graduate students had volunteered within 1 week, and 31% were working in nine emergency pandemic departments [25]. Medical students from Years 2 to 6 in Basel, Switzerland, were invited to be volunteers and they responded promptly. Medical students worked in two shifts of 12 students each, for a total of 936 shifts [40]. 

A survey of third to fifth-year medical students from Hamburg, Germany, was conducted online. According to the findings, 68 students signed up, but only 25.0% were called to work. About half of the students, 47.1%, were involved in phone consultations or data management 29.4%. In the hospital, five students, 29.4%, volunteered for ambulance services, and 11.8% worked in general practices [23]. Another example we noticed was at the University Medical Center Mainz, Germany, where the medical students’ support was urgently needed when the CoronaPreventMainz (CPM) study was initiated in April 2020. All medical students enrolled in the CPM study were asked to complete a survey to assess their competence for a commitment to the pandemic as well as the influence on their abilities and attitudes. The large majority of students (87.3%) believed that students should be volunteer workers during this crisis, and 90.5% felt they were helping the COVID-19 pandemic. The majority of students (80.6%) even stated that their practical abilities had improved. Self-infection fear was modest (7.9%), and “overextending situations” occurred in just 3.2%. In fact, only around 19.4% of students felt prepared for the SARS-CoV-2 pandemic at the end of medical school, and 67.7% required further training [17].

Allowing students inside the clinic raises the potential of contamination and, as a consequence, the risk of disease transmission, mainly because of the lack of personal protective equipment, which has been highlighted since the beginning of the epidemic [41]. Concerns about student activity in the hospitals were exacerbated by the lack of personal protective equipment (PPE). To save personal protective equipment, New York-Presbyterian, and other hospitals demanded that surgical gowns and masks be worn only by those who were absolutely necessary [20].

Another interesting study looked into the volunteers’ perceptions of the conditions, safety, costs, and benefits of participating in volunteer programs at the Poznan University of Medical Sciences in Poznan, Poland. The majority of respondents noted an improvement in their soft skills, with 65.82% agreeing that they learned new and fascinating things [16]. However, the benefits of volunteering may exceed the risks, with students having the chance to learn new talents, social and organizational skills, stress management techniques, and professional growth. Interprofessional service-learning allows students to help patients, develop their clinical knowledge, intensify their professionalism, apply interpersonal and communication skills, participate in “rapid-cycle practice-based learning and improvement”, and make a real contribution to systems-based practice. The present pandemic environment also gives a chance to observe the medical system’s reaction under circumstances of a health crisis, such as resource management, given a lack of staff and material limitations. COVID-19 is an ever-changing scenario, full of opportunities to develop a professional identity for what it means to be a doctor [16,20].

In our study, international students were more involved in volunteering activities in hospitals, probably following the needs and facilities offered in their country of origin, since they spent more time there during the pandemic. On the other hand, women were more involved in health education activities.

There are several limitations of this study. The study included only last-year medical students from one medical faculty from Romania. As other studies using online questionnaires also showed, we had a response rate of 39% of students invited to participate in the survey. On the other hand, an exploratory questionnaire was used, which did not allow for more in-depth analyses.

## 5. Conclusions

Since the onset of the COVID-19 pandemic, the entire clinical, scientific, and academic medical system has learned several lessons.

Fear of infection resulted in the introduction of societal constraints, demanding the development of solutions to let clinical, scientific, and academic activities continue safely. The most convenient solution was to relocate all face-to-face meetings to an online environment. Furthermore, the pause in the development of scientific projects has resulted in the rethinking of many studies, as well as the changing of the topic in cases where the research hypothesis could not be tested or the experiments could not be carried out. As a result, this pandemic has affected the whole field of research, particularly medical students, who have faced incertitude and insecurity over the proper conduct of research due to a lack of experience. In addition, the crisis in the medical system due to lack of staff has contributed massively to the formation of student volunteer organizations among medical students. Several studies have reported the availability and interest shown by students to participate in such activities. Following their voluntary work, the students reported that they had developed some clinical skills as well as a lot of new information. Due to the suspension of clinical rotations during the lockdown, many students found that volunteering was a useful way to practice [16,17,20].

This is the first study from Romania and one of the few from Europe that presents data regarding the influence of the COVID-19 pandemic on research and volunteering activities among medical students.

The study sample consisted of medical students from their last year of study from both the Romanian section as well as the international section, comprising English and French sections. Hence the data reflect the difficulties of medical students from different countries who study in Romania in terms of finishing their graduation thesis, but also their involvement in different types of volunteering activities. The differences between the two sections concerning some issues might be explained by the fact that international students were confronted with traveling restrictions and spent more time in their country of origin, influencing their research capacity but also offering them different environments for building resilience skills, including volunteering activities.

## Figures and Tables

**Table 1:** Impact of COVID-19 pandemic regarding research activities conducted by students in their final year.

	Total *n* = 232 %	Romanian Section *n* = 149 %	English and French Section *n* = 83
COVID-19 pandemic affects research activities	65.5	62.4	71.1
Changing the subject of the thesis	9.5	7.4	13.3
Review of the literature instead of data collection	22.8	16.1	34.9
Difficulty in contacting participants	24.6	22.8	27.7
Difficulty in developing research activities in the laboratory	12.1	10.1	15.7
Difficulty in developing research activities in the hospital	35.3	30.9	43.4
Difficulty in developing research activities in the community	6	4.0	9.6
Difficulty in data centralization and analysis	13.4	14.8	10.8
Difficulty in communication with the thesis coordinator	31.5	30.2	33.7

**Table 2:** Impact of COVID-19 pandemic regarding volunteering activities conducted by students in their final year.

	Total *n* = 232 %	Romanian Section *n* = 149 %	English and French Section *n* = 83 %
Conducting volunteering during COVID-19 pandemic	22	20.8	24.1
Involvement in the public health directorate	2.6	2.7	2.4
Volunteering at the hospital	6.9	2.7	14.5
Volunteering in the follow-up of patients with general practitioners	3	2	4.8
Volunteering in the transport of medicines/food	3.4	4	2.4
Volunteering in vaccination institution	2.2	1.3	3.6
Participate in health education activities	5.2	5.4	4.8

**Table 3:** Factors associated with the impact of the COVID-19 pandemic on research and volunteering activities.

	Section		Gender	
	OR (CI)	*p*	OR (CI)	*p*
COVID-19 pandemic affects research activities	1.480 (0.830–2.641)	>0.05	1.160 (0.671–2.007)	>0.05
Changing the subject of the thesis	1.917 (0.793–4.635)	>0.05	0.832 (0.344–4.033)	>0.05
Review of the literature instead of data collection	**2.797 (1.493–5.341)**	**<0.01**	0.736 (0.397–1.364)	>0.05
Difficulty in contacting participants	1.297 (0.701–2.397)	>0.05	1.058 (0.576–1.918)	>0.05
Difficulty in developing research activities in the laboratory	1.659 (0.748–3.681)	>0.05	0.571 (0.258–1.262)	>0.05
Difficulty in developing research activities in the hospital	**1.715 (1–2.991)**	**0.05**	1.258 (0.725–2.183)	>0.05
Difficulty in developing research activities in the community	2.542 (0.851–7.597)	>0.05	0.938 (0.314–2.795)	>0.05
Difficulty in data centralization and analysis	0.702 (0.307–1.305)	>0.05	**3.378 (1.328–8.521)**	**<0.05**
Difficulty in communication with the thesis coordinator	1.177 (0.663–2.089)	>0.05	1.306 (0.739–2.038)	>0.05
Conducting volunteering during COVID-19 pandemic	1.208 (0.637–2.229)	>0.05	1.247 (0.658–2.364)	>0.05
Volunteering in the public health directorate	0.895 (0.160–4.994)	>0.05	1.424 (0.256–7.937)	>0.05
Volunteering at the hospital	**4.868 (1.651–14.352)**	**<0.01**	0.604 (0.224–1.627)	>0.05
Volunteering in the follow-up of patients with general practitioners	2.464 (0.538–11.238)	>0.05	1.794 (0.341–9.445)	>0.05
Volunteering in the transport of medicines/food	0.558 (0.116–2.984)	>0.05	1.183 (0.276–5.074)	>0.05
Volunteering in vaccination institution	2.756 (0.451–16.839)	>0.05	1.060 (0.174–6.190)	>0.05
Participation in health education activities	0.892 (0.260–3.057)	>0.05	**8.360 (1.361–65.883)**	**<0.05**

Section is coded as 0—Romanian, 1—international section comprising English and French sections. Gender is coded as 0—boys, 1—girls. Number in bold indicates statistically significant results.

## Data Availability

Data can be obtained on justified cases from the corresponding author.
